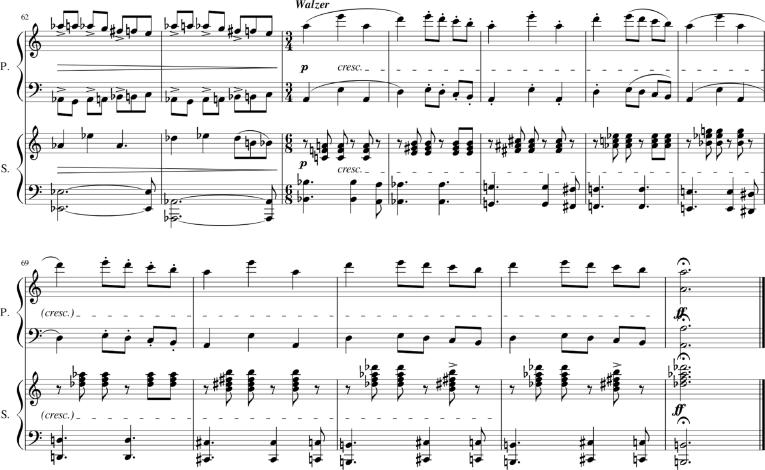# Eye gaze as a means of giving and seeking information during musical interaction

**DOI:** 10.1016/j.concog.2019.01.002

**Published:** 2019-02

**Authors:** Laura Bishop, Carlos Cancino-Chacón, Werner Goebl

**Affiliations:** a Austrian Research Institute for Artificial Intelligence (OFAI), Vienna, Austria; b Institute of Computational Perception, Johannes Kepler University Linz, Austria; c Dept. of Music Acoustics, University of Music and Performing Arts Vienna, Austria

**Keywords:** Coordination, Visual interaction, Communication, Eye gaze, Body gestures, Joint action, Attention

## Abstract

During skilled music ensemble performance, a multi-layered network of interaction processes allows musicians to negotiate common interpretations of ambiguously-notated music in real-time. This study investigated the conditions that encourage visual interaction during duo performance. Duos recorded performances of a new piece before and after a period of rehearsal. Mobile eye tracking and motion capture were used in combination to map uni- and bidirectional eye gaze patterns. Musicians watched each other more during temporally-unstable passages than during regularly-timed passages. They also watched each other more after rehearsal than before. Duo musicians may seek visual interaction with each other primarily, but not exclusively, when coordination is threatened by temporal instability. Visual interaction increases as musicians become familiar with the piece, suggesting that they visually monitor each other once a shared interpretation of the piece is established. Visual monitoring of co-performers’ movements and attention may facilitate feelings of engagement and high-level creative collaboration.

## Highlights

• Periods of temporal instability prompt duo musicians to monitor each other visually.
• Bidirectional gaze between musicians occurs at piece onset and in the final beats.
• Duo musicians watch each other increasingly as they become familiar with a piece.
• Patterns of uni- and bidirectional gaze across duo performances are similar.
• Gaze behaviour reflects leader/follower roles implied by the score.

## 1 Introduction

When people interact, eye gaze serves as a means of giving, as well as obtaining information. The direction of a person’s gaze can indicate their focus of attention – which might be on another person, a salient environmental stimulus, or the direction they intend to move. Indeed, some researchers have hypothesized that the human eye evolved its current structure (specifically, the contrast between the coloured iris and white sclera) because people benefitted from the ability to track each other’s gaze direction (Tomasello, Hare, Lehmann, & Call, 2007). When eye gaze is directed towards another person, it often indicates an intention to interact. Imaging studies have shown that direct eye gaze activates the “social brain”, a network of structures involved in human communication and social interaction (Senju & Johnson, 2009). It follows that eye gaze can be a valuable means of communicating during joint action tasks, such as playing team sports or performing with a music ensemble.

Our study assessed the communicative functions of eye gaze during music ensemble performance, a form of joint action that requires precise and multi-layered coordination between participants. Individual note onsets and offsets, as well as expressive nuances (e.g., changes in patterns of loudness and articulation) have to be aligned in time, even though the information provided by the score (if there is one) may be limited and subject to interpretation.

During ensemble performance, visual communication between musicians is secondary in importance to auditory communication, but can become more relevant when performers are uncertain of each other’s interpretations (Bishop & Goebl, 2015). Prior research has shown that temporal instability, such as happens at piece entrances, long pauses, and sudden tempo changes (Bishop and Goebl, 2017, Davidson, 2012, Kawase, 2014a, Kawase, 2014b), and disruptions to the clarity (e.g., loudness) of inter-performer audio feedback (Fulford, Hopkins, Seiffert, & Ginsborg, 2018) can prompt an exchange of visual cues. These are sometimes deliberately integrated into a performance plan across rehearsals (Williamon & Davidson, 2002).

The current study was designed to build on these findings by examining patterns of uni- (one-way) and bidirectional (two-way or mutual, see Emery (2000)) eye gaze as they unfolded across the course of piano and clarinet duo performances. Our aim was to identify the conditions that prompt performers to interact visually. Based on theoretical understandings of the mechanisms that support musical interaction (discussed in the next section), we hypothesized that differences in playing conditions would encourage different degrees of visual interaction.

### 1.1 Functions of eye gaze in musical interaction

We defined two functions of interperformer eye gaze, both of which we expected to see evidence of during duo performance. Our definitions of these functions are theoretically-motivated, which we describe below, and demonstrate our attempt to reconcile the sometimes-contrasting predictions that derive from different perspectives on musical interaction. By defining these functions and seeking measurable behavioural evidence of them, we aimed to highlight the variable nature of musical interaction and shed some light on which processes support duo performance as playing conditions change.

Our underlying argument was that, as a performance unfolds, musicians are at some moments in time drawn to engage deliberate planning and communication processes to ensure that coordination is achieved or maintained. They might exchange gestural cues, facial expressions, or make audible inhalations to facilitate synchronization following a long pause, for example. Outside of these moments, they allow coordination to “emerge” from the subtle, pre-reflective accommodations that they make to each other’s audiovisual feedback. Of course, successful emergent coordination at note and expressive levels is contingent on performers attending closely to each other and their combined musical output.

#### 1.1.1 Engagement-driven eye gaze

Engagement-driven eye gaze is used by musicians to monitor each other’s attention and engagement in the joint performance task, as well as to indicate their own attention and engagement. This information can be provided by musicians’ gaze direction, facial expressions, and body movements. For example, eye gaze directed towards the observing co-performer might indicate a focus on the interperformer interaction, while a particular pattern of body sway might indicate focus towards an aspect of expression. It is important for musicians to be aware of each other’s focus of attention (e.g., whether it is directed to the score, or to the co-performer, signalling an intent to interact, or elsewhere, signalling potential distraction), because their attentiveness relates to how responsive they are likely to be to subtle fluctuations in each other’s audiovisual signals.

In the context of the current study, evidence of engagement-driven gaze would include the occurrence of bidirectional gaze that does not occur exclusively at moments of temporal ambiguity (though perhaps at other structurally significant points; e.g., piece ending). Bidirectional gaze between duo performers allows visual signals to flow in both directions and signifies that both performers are monitoring each other. In particular, bidirectional gaze that occurs during periods of temporal stability, when visual communication is not necessary for performers to maintain coordination (Bishop and Goebl, 2015, Fulford et al., 2018, Kawase, 2014a), is suggestive of performers’ attempts to confirm each other’s attention.

#### 1.1.2 Intention-driven eye gaze

Intention-driven eye gaze is used by musicians to communicate their individual intentions and learn their co-performers’ individual intentions. By “individual intentions”, we refer to action-based plans that are known to one performer, but not necessarily to others (e.g., a performer may intend to play a particular passage softly). These are to be distinguished from “shared intentions”, which are action-based plans that overlap between performers (e.g., all performers may intend to play the passage softly). In particular, musicians are known to communicate visually to align their intended timing, and their cueing gestures often take the form of an exaggerated nod (Bishop & Goebl, 2017).

In the context of the current study, evidence of intention-driven gaze would include the occurrence of unidirectional gaze at moments of temporal ambiguity (e.g., piece onset), particularly when directed from follower to leader. Musical passages with ambiguously or imprecisely notated timing may be interpreted differently by different performers; thus, uncertainty about each other’s interpretation might prompt performers (especially those in a leading role) to communicate their own intentions or (in the case of followers) seek out information about their co-performer’s intentions. In such cases, the leader might watch the follower while performing a gestural cue to ensure that the follower is paying attention. While the follower’s gaze is intention-drive, the leader’s follower-directed gaze is engagement-driven.

In the next section, we discuss the theoretical underpinnings of these definitions.

### 1.2 Cognitivist and enactive perspectives on musical interaction

Some forms of joint action involve deliberate collaboration between group members with the aim of achieving a particular goal. Tasks such as coordinating a pass between people on the soccer field, lifting a table into a truck, or playing a piano duet according to a particular style fall into this category. In the literature, this is referred to as planned coordination, and defined in contrast to emergent coordination, which occurs unintentionally as people make (largely) automatic accommodations to low-level features in each other’s audio and visual signals (e.g., people who are walking together may find themselves stepping in sync; van der Wel, Sebanz, & Knoblich, 2016). It is important to note that planned and emergent coordination are not mutually exclusive, and during ensemble performance, occur in parallel.

According to the cognitivist account of joint action, during planned coordination, shared intentions and shared attention to the task are necessary for group members to achieve their desired goal (Keller, 2014, Knoblich and Sebanz, 2008). The term intentions refers broadly to a dynamic, action-based process of representing or anticipating upcoming actions or events. This process is flexible – intentions are constantly evolving as performers monitor their own and their co-performers’ output. Flexibility in action planning is important because it allows group members to compensate for variability in each other’s performance, which might arise because of errors, environmental disturbances, or the spontaneous introduction of new ideas (Loehr et al., 2013, Noy et al., 2011; for an overview see Bishop, 2018). Shared intentions comprise overlapping, but not identical, representations of how individual efforts should contribute to the overall outcome. In essence, each member of the group understands their own contribution in the terms of how it will combine with others’ contributions (Gallotti & Frith, 2013).

Another perspective on the processes underlying interpersonal coordination derives from embodied, enactive, and distributed cognition and dynamical systems theories (Geeves and Sutton, 2014, Leman and Maes, 2014). By this account, entrainment between individuals occurs spontaneously as a result of low-level sensory (auditory and visual) couplings, or accommodation (Maes, 2016). For ensemble musicians, this process potentially enables coordination at both local (note) and global (expressive) levels. Some researchers argue that shared intentions, as defined by cognitivists, may not be needed for musicians to coordinate their performance in this way, because coordination can arise independently of such top-down control as musicians engage in cycles of small-scale responses to the joint output (Schiavio & Høffding, 2015). Evidence of coordinated improvised performance in the absence of preplanned structures has been observed in both music (Canonne & Garnier, 2015) and dance domains (Kimmel, Hristova, & Kussmaul, 2018), suggesting that emergent coordination can indeed occur in artistic contexts.

MacRitchie, Varlet, and Keller (2017) propose that representational and entrainment processes may run in tandem, with one or the other exerting a dominant influence on behaviour depending on the performers’ familiarity with each other’s roles in the performance. For example, in the early stages of rehearsal, ensemble members may be unsure of how each other will interpret an unfamiliar piece, and representational processes may dominate as they try to establish a shared interpretation. Jointly rehearsing a new piece was the task given to participants in the current study. As they rehearsed, their familiarity with each other’s playing increased and they established shared intentions for how the piece should sound, potentially reducing the likelihood that they would have to communicate individual intentions in order to coordinate their performance.

One of the specific hypotheses we tested was whether performers would spend less time watching each other as the rehearsal progressed. Such a finding would suggest that gaze is largely intention-driven during the early stages of rehearsal, and decreases once performers are more certain of how each other intends to play. The opposite finding – an increase in partner-directed gaze across the rehearsal (already observed in trios; see Vandemoortele et al., 2018) – would suggest that familiarity with the music and with a shared, practiced interpretation encourages the use of engagement-driven gaze.

### 1.3 Influences on gaze behaviour

With these perspectives in mind, our study considered the possibility that eye gaze serves multiple communicative functions during ensemble performance – specifically, that it is primarily a means of affirming co-performers’ attention and engagement, and secondarily a means of ascertaining co-performers’ intentions. Engagement-driven gaze, which allows for the exchange of subtle attention, expressive, and movement-related signals, may help to support entrainment processes. Intention-driven gaze, in contrast, which reflects a drive to exchange intentions that would not otherwise be shared, may help to support representational processes.

Much of the research on visual interaction during ensemble performance focuses on musicians’ body gestures. The questions addressed include how timing information might be encoded in these gestures, and how well other musicians are able to synchronize their own gestures with those they observe (Bishop and Goebl, 2017, Bishop and Goebl, 2018, Wöllner and Cañal-Bruland, 2010, Wöllner et al., 2012). The assumption behind these studies is that musicians choose to attend to each other’s gestures to obtain information that might facilitate coordination. In particular, a performer who is assuming a “follower” role might monitor the performer who is assuming a “leader” role for gestural cues.

The current study investigated whether patterns of partner-directed gaze relate to performers’ assumption of leader/follower roles. Research has already shown that leader-follower relationships emerge among ensemble members (Badino et al., 2014, Timmers et al., 2014), and that these relationships are reflected in the amount of time that performers spend looking towards one another – when leader/follower roles are assigned, followers look more towards leaders than vice versa (Kawase, 2014a). Many factors contribute to the emergence of leader-follower relationships in musical duos, some relating to genre conventions (e.g., in Western classical music, the performer playing the solo or melody typically leads) and others relating to social factors (e.g., relative skill levels of the performers, personality traits, etc.).

As a contrast to previous studies in which leader/follower roles were assigned, we tested specifically whether leader-follower relationships that are implied by piece structure (i.e., melody vs. accompaniment assignments) influence gaze patterns. If performers were to watch their partner more when following (playing accompaniment) than when leading (playing the melody), it would suggest the use of gaze as a means of obtaining information about the leader’s intended interpretation. Our hypothesis was that leader/follower relationships might be reflected in gaze behaviour during some parts of a performance (e.g., during periods of high temporal instability), but not during others (e.g., during periods with regular timing), indicating an interaction with musical structure.

Musicians’ tendency to visually monitor each other’s attention and engagement was likewise expected to rise and fall across the course of a performance. To assess this tendency, we tested whether performers tended to look more towards their partners’ face than towards their partners’ bodies or instruments. In cases where instrument motion is likely to be informative (e.g., during clarinet performance), a preference for looking at the face would suggest that gaze is being used to communicate information about attention. We also considered how often partner-directed gaze was bidirectional, rather than unidirectional. During moments of bidirectional gaze, information flows both ways simultaneously – from primo to secondo and from secondo to primo – which, in addition to giving both performers visual access to each other’s body gestures and facial expressions, communicates to each performer that they are the focus of their partner’s attention.

Direct eye gaze is treated specially by the human brain, affecting the way people perceive others and judge their mental capabilities. Faces with direct gaze are judged as belonging to people with more sophisticated mental faculties than faces with averted gaze, for instance, possibly because direct gaze acts as a cue to impending social interaction and prompts people to expend more effort in considering others’ internal experiences (Khalid, Deska, & Hugenberg, 2016). In certain types of interaction, gaze is drawn to others’ eyes and faces. For example, deaf viewers have been found to focus mostly on signers’ faces when watching sign language video clips, processing hand and arm movements largely with their peripheral vision (Muir & Richardson, 2005). Face-directed gazing during signing allows the viewer to detect facial expressions and lip movements, and it allows the signer see that the viewer is paying attention. Along the same lines, studies of spoken conversation have shown that speakers expect listeners to confirm their attention through auditory or visual backchannelling, and will periodically pause and look towards their listeners, seeking such a response (Bavelas, Coates, & Johnson, 2002). Direct eye gaze may likewise contribute to backchannelling during music ensemble performance.

More broadly, in non-musical forms of interaction, perceived gaze direction has been shown to help people track each other’s attention. A study by Khoramshahi, Shukla, Raffard, Bardy, and Billard (2016) provides a clear example. In this study, human participants collaborated with an avatar on the “mirror game”, a task in which two participants, while facing each other directly, move a pair of sliders along horizontal tracks to produce creative but synchronized patterns of motion. Participants were told to follow the avatar’s lead. When the avatar provided anticipatory gaze cues (i.e., began looking in the direction of its next movement just before initiating the movement), leader-follower synchronization was better than when the avatar visually tracked its hand movements (i.e., always looked directly at its moving hand). Anticipatory gaze cues, therefore, allowed participants to account for the avatar’s intentions in their own action planning. We hypothesized that ensemble musicians monitor each other’s gaze direction when it is possible to do so, possibly because tracking fluctuations in each other’s attention – as well as receiving backchannelling signals – helps to support emergent coordination.

### 1.4 Current study

This study investigated patterns of eye gaze during duo piano and clarinet performance. Motion capture and eye tracking were used to map musicians’ body gestures and gaze patterns as they rehearsed and performed a new duet piece. The piece was composed specifically for this study and contained a number of potential challenges for coordination (see Section 2.3). Duos recorded four full performances of the piece between periods of free joint rehearsal. The final performance was given with no visual contact between performers; the other performances and free rehearsal periods were completed under normal visual contact conditions. Eye gaze data were analysed to determine when and how much musicians looked towards their co-performers. Motion data are presented in Bishop and Goebl (submitted for publication).

Patterns of partner-directed eye gaze were expected to distinguish conditions that encourage the use of visual interaction for confirming co-performers’ attention and engagement (engagement-driven gaze) from the use of visual interaction for communicating individual intentions (intention-driven gaze). To this end, we assessed the potential effects of rehearsal, leader/follower relations, and musical structure on the amount of time that musicians spent watching their partners. It should also be noted that glances from one performer to another can be multifunctional, enabling a performer to gain information about their co-performer’s attention and intentions simultaneously. For this reason, the manipulation of musical structure was particularly important, as we expected temporal uncertainty resulting from imprecisely notated timing to encourage intention-driven gaze in particular. Engagement-driven gaze, in contrast, could occur at any time, and was not expected to account for heightened tendencies for partner-directed gaze at moments of temporal instability.

As evidence of engagement-driven gaze, periods of bidirectional gaze were expected to occur throughout performances. Musicians were expected to look at each other even during temporally stable passages and during performances that followed the rehearsal period. For clarinet duos, partner-directed gaze was also expected to target the face more often than the instrument/body, even though clarinet bell movements are known to carry information relevant to expression and timing (Wanderley, Vines, Middleton, McKay, & Hatch, 2005).

As evidence of intention-driven gaze, musicians were expected to look towards their partner when uncertain of their partner’s intentions – for example, at piece onset, during periods of temporal instability in the music, and at the start of the rehearsal period. They were also expected to look more towards their partner when playing an accompaniment passage (i.e., when following) than when playing a melody passage (i.e., when leading).

## 2 Methods

### 2.1 Participants

Forty-two musicians participated in the experiment, 20 pianists and 22 clarinettists, all of whom perform professionally. Pianists (age M=28.3 years; 14 female) reported an average of 21.6 years of training (SD=7.3) and 18.8 performances per year (SD=16.7); clarinettists (age M=24.0 years; 13 female) reported an average of 15.0 years of training (SD=4.2) and 53.7 (SD=53.2) performances per year. All participants provided informed consent and received a small compensation, and the study received prior approval by the University of Music and Performing Arts Vienna ethics committee.

### 2.2 Design

The effects of three main within-subject independent variables were tested: (1) piece structure, (2) rehearsal time, and (3) leader/follower roles. The structure of the duet piece is outlined below (see Section 2.3). As part of the analysis procedure, the piece was segmented into windows of interest, and performers’ behaviour was compared between windows (*piece structure* variable). The three performances recorded with normal visual contact were given before, partway through, and immediately following a rehearsal period (*rehearsal time* variable). Instrument pairing (piano-piano or clarinet-clarinet) was manipulated between subjects. Our primary dependent variable was the amount of time (i.e., percentage of recorded gaze samples) that performers spent looking at their partner. For clarinettists, as a secondary dependent variable, we also compared the percentage of time dedicated to face-directed versus body/instrument-directed gaze.

### 2.3 Stimuli and equipment

**Fig. 1:**
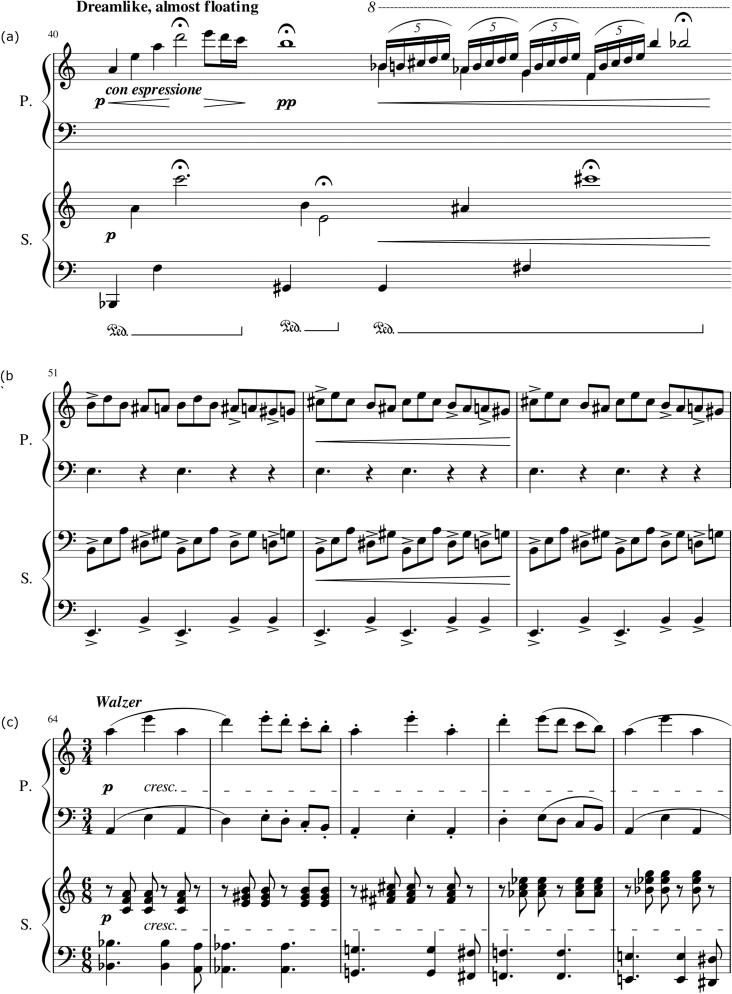
Three one-line excerpts from the piano version of the stimulus piece. Line (a) is the start of the unmetered section, line (b) is taken from a section in 5 + 7/8 meter, in which the primo and secondo have to synchronize accent patterns, and line (c) is the start of the final section, in which the main theme returns in the primo, and the primo and secondo have different meters.

A duet was composed for the experiment by the second author, a pianist and composer with 14 years of musical training. Some excerpts from the piece are given in Fig. 1. Though primo and secondo parts were not technically very difficult, the piece was meant to be challenging for a duo to coordinate. There were several changes in meter and tempo, some unusual meters (e.g., 5 + 7/8), one unmetered section, and sections with accent patterns that the primo and secondo were intended to synchronize. The piece was initially written as a piano duet, then arranged for two clarinets with the aid of a professional clarinettist. See the Appendix for the full piano score.

**Fig. 2:**
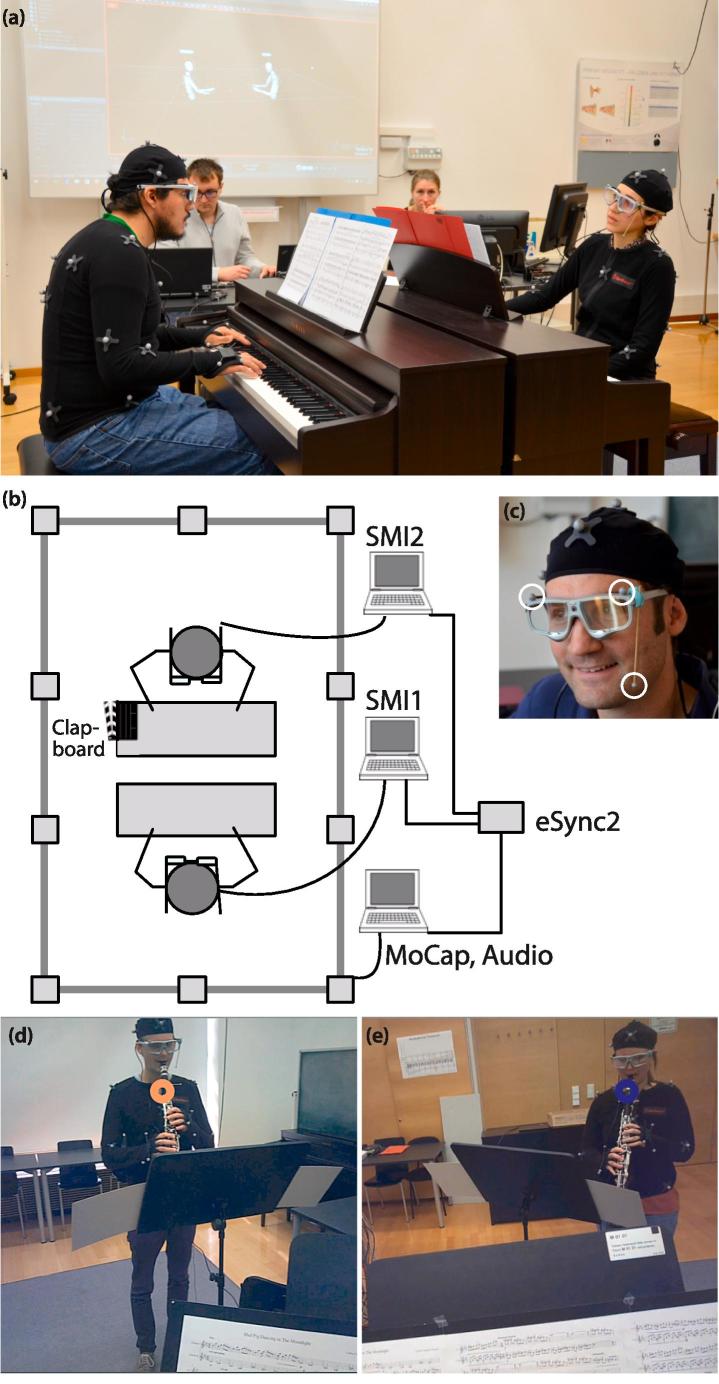
Experimental procedure. (a) A piano duo recording a performance (photo taken during a pilot test; note that for the experiment, the projector screen was switched off during recordings and landscape-oriented scores were used); (b) a diagram of the recording and data synchronization set-up involving a central clock and three computers; (c) eye tracking glasses fitted with markers for detection by the motion capture system (circled in white); and (d–e) frames captured by the eye tracking glasses worn by either performer during a clarinet duo performance (orange/blue circles indicate viewers’ gaze positions). (For interpretation of the references to color in this figure legend, the reader is referred to the web version of this article.)

Performers wore SMI ETG 2 wireless glasses, through which eye gaze was tracked at 120 Hz (Fig. 2). These glasses track gaze with up to 0.5° accuracy over all distances (SensoMotoric Instruments (SMI), 2017). The glasses cannot be worn over normal prescription glasses, which create too much interference; however, magnetic snap-on corrective lenses were available for participants requiring a distance correction (within the prescription range of −4.0 to +4.0). The glasses were fitted with markers for detection by the motion capture system. Two markers were placed on top of the glasses frame, at the corner of each lens. The third marker was placed on a small stick, which was attached to the glasses frame with dental putty and extended down from the left arm of the glasses.

A 10-camera (Prime 13) OptiTrack motion capture system was used to track performers’ upper body movements, recording at a rate of 240 frames per second. Each performer was fitted with 25 reflective markers. Additional markers (3) were fixed to the eye tracking glasses, the corners of the music scores (4), and, in the case of clarinet duos, both performers’ instruments (4). Eye gaze data for the two performers were collected with separate laptops, both of which were connected to an OptiTrack eSync 2 device (see Fig. 2), which transmitted TTL triggers from the motion capture software to the eye tracking software at the start and end of each motion capture recording. These triggers were logged in the gaze data files as a numerical variable, allowing us to retrospectively align eye gaze and motion capture data by trimming data files to exclude observations outside the trigger range.

Pianists performed on Yamaha Clavinovas, from which audio and MIDI data were collected via a Focusrite Scarlett 18i8 sound card and recorded on separate tracks in Ableton Live. Clarinettists performed on their own instruments, and their audio was likewise recorded in separate tracks, using DPA d:vote 4099 clip-on microphones. A clapboard was placed within range of an additional room microphone and within view of the OptiTrack and SMI cameras and struck once at the start and end of each recording. This provided a synchronization stimulus, recorded on all devices, that allowed us to align audio/MIDI with motion capture and eye gaze data.

### 2.4 Procedure

At the start of the session, performers were presented with the piece and randomly assigned either the primo or secondo part. They were told that our aim was to investigate performer interaction during rehearsal of unfamiliar music. Duos were asked to practice the piece in preparation for making some high-quality recordings at the end of the session. They were not required to memorize the music.

**Fig. 3:**
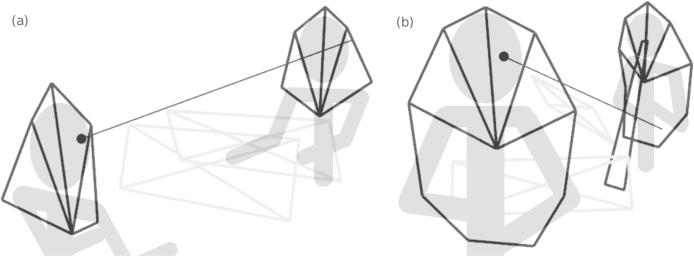
Visualizations of gaze vectors intersecting (a) a pianist partner’s face and (b) a clarinettist partner’s body. The black dots indicate the position of the gaze vector origins, and the line extending from each dot represents the gaze vector. Grey rectangles indicate the position of the performers’ scores, and four adjoining triangles outline the performers’ faces. In (b), the partner’s clarinet (long black rectangle) and both performers’ upper bodies are also outlined.

Performers were positioned so that they faced each other, roughly 1.5 m apart. Clarinettists played standing without any specific instruction on how to orient themselves, so they had more freedom to move around than did pianists (e.g., see how the clarinettists’ scores ended up at an angle to each other in Fig. 3b).

An initial (sight-read) performance was recorded first, with performers encouraged to ignore errors and play through as much of the piece as possible without stopping. This performance was followed by up to 20 min of free joint rehearsal. (Some duos required more practice time than others. To prevent duos from reaching peak performance by the end of the first rehearsal, duos who were progressing quickly were asked to stop when the experimenters could hear that they still had some passages to work out.) A second full performance was then recorded, followed by up to 20 more minutes of rehearsal. Finally, two “polished” performances were recorded, one with visual contact between performers, and then one without (always in that order). Musicians also completed a short questionnaire on their musical background and answered a few debriefing questions about their perceptions of the experiment.

### 2.5 Analysis

#### 2.5.1 Note onsets

Note onsets in clarinet performances were identified manually using waveform and spectrogram information. For piano performances, MIDI data collected from the Clavinovas were matched to the score using the score-performance matcher developed by Flossmann, Goebl, Grachten, Niedermayer, and Widmer (2010). This system pairs performed pitches with score notes based on pitch sequence information, disregarding the timing. Incorrectly performed pitches (additions and substitutions) are omitted, so the resulting matched performance profile includes only correctly performed notes.

#### 2.5.2 Eye gaze

Vectors indicating direction of gaze for the right eye of each participant were exported from the eye tracking software. For each sample of eye gaze data, a “gaze target” was identified. For pianists, gaze targets took one of three values, indicating whether the participant was looking towards the score (“score”), the other performer’s face (“face”), or elsewhere in the room, including towards the piano keys (“other”). For clarinettists, gaze targets could take an additional value, indicating whether the participant was looking at the other performer’s upper body/instrument (“instrument”) (Pianists were only able to see each other’s faces over their scores, and clarinettists’ views of each other’s lower bodies were largely occluded by the music stands).

It was necessary to allow for small degrees of imprecision in the gaze vector coordinates obtained by the glasses resulting from calibration errors, which sometimes occurred over the course of the 1-h recording session (e.g., if participants bumped or shifted the glasses following the initial calibration step). To account for such errors, we expanded the region of each gaze target by a small amount so that gaze vectors that fell slightly outside a region would be counted as intersecting that region. For all duos, markers indicating the sides of the head were adjusted outwards by 1.4 times the length of the vector between them; the same was done for the markers indicating the left and right shoulders, and for the markers outlining the clarinets. The assumption here was that participants were unlikely to spend time watching anything but each other, the score, and their instruments, given their unfamiliarity with the music. Thus, a participant who appeared to be staring at a spot on the wall just to the left of their co-performer’s head was most likely actually looking at their co-performer’s face.

We developed an automated procedure for identifying gaze targets, which involved remapping gaze vector coordinates into the motion capture space. As a first step, motion capture and eye tracking data were temporally aligned using the recorded trigger values (see Section 2.3), and linear interpolation was used to resample gaze vector profiles at 240 Hz, the sampling rate of the motion capture recordings.

Next, a 4×4 rotation matrix was used to rotate all points in the motion capture space around the origin of the gaze vector (i.e., the position of the right eye). The x component of the matrix corresponded to the axis defined by the pair of markers on top of the glasses frame, the y component corresponded to the axis defined by the markers on top of and extending below the frame on the left side of the glasses, and the z component corresponded to the axis defined by the normal vector to the plane containing the three glasses markers. Gaze vector coordinates were then added to the rotated and transformed gaze vector origin.

In most cases, a small correction to the direction of the gaze vector was needed to compensate for imprecision in the placement of markers on the glasses and estimation of the gaze vector origin. A secondary 3×3 rotation matrix was constructed, and one set of (x, y, and z) angle values was entered manually for each recorded performance, based on visual comparison with the video and gaze marker display viewable within the eye tracking software.

Finally, we tested whether the corrected gaze vector intersected with the score, the other performer’s face, or, for clarinettists, the other performer’s body or instrument. Fig. 3 provides visualizations of these tests. Scores were defined as four-sided polygons using markers that had been placed in the corners. Performers’ faces were defined as four adjacent triangles using markers located on the top and sides of the head, on the chest, and on the shoulders. Clarinettists’ upper bodies were designated by five adjacent triangles, using markers located on the shoulders, chest, upper arms, and waist. Clarinets were defined as four-sided polygons, using markers placed near the mouthpiece and near the bell. A sample video showing the gaze behaviour of two pianists during a final performance can be viewed in the supplementary files.

#### 2.5.3 Missing data

Eye gaze data are not reported for two pianists and two clarinettists, for whom we were unable to get reliable recordings. (The glasses are sometimes unable to track pupil position; for example, if the participant is squinting, has swelling around the eyes, or has a flatter nose/narrower eyes than are typical for people of European descent.) Motion capture data was also lost for one clarinet duo and partially lost for a second clarinet duo, due to file corruption.

## 3 Results

### 3.1 Differences in temporal stability across piece sections

One of our primary hypotheses was that the percentage of time that performers spent watching each other would increase in passages where timing information was loosely-defined by the score (and the chances of performers’ interpretations diverging was greater) and decrease in passages with less temporal ambiguity. We refer to passages with loosely-defined timing information as “temporally unstable”, because variability in performance timing was expected to increase. To confirm, analyses of (1) tempo variability and (2) primo-secondo note asynchronies were conducted for piano duo performances. The analyses were only run on the MIDI data acquired from pianists since note onsets in clarinet performances were identified manually, and therefore less precise.

The piece was divided a priori into sections, with boundaries placed where they made musicological sense (i.e., when meter and texture changed). The first 8 bars (“entrance”), the final 10 bars (“ending”), and the unmetered section from the middle of the piece (“unmetered”) were expected to invoke greater temporal instability than the other, more regularly-timed sections (“regular”).

**Fig. 4:**
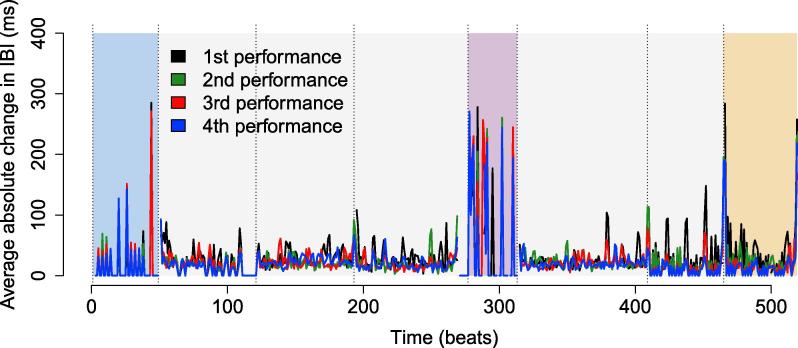
Absolute change in IBI per beat of the piece, averaged across piano duos. Dotted vertical lines indicate points of metrical change, and the sections shaded blue, pink, and yellow indicate entrance, unmetered, and ending sections, respectively – all other sections were regularly timed. (For interpretation of the references to color in this figure legend, the reader is referred to the web version of this article.)

Interbeat intervals (IBIs) were taken as a measure of performance tempo using averaged primo-secondo note onsets. As a measure of within-performance tempo *variability*, the series of IBIs was differenced once (Fig. 4). The resulting series indicated how IBI durations changed across the course of the piece: high values reflected large note-to-note changes in tempo, while small values reflected a more consistent tempo over time.

**Fig. 5:**
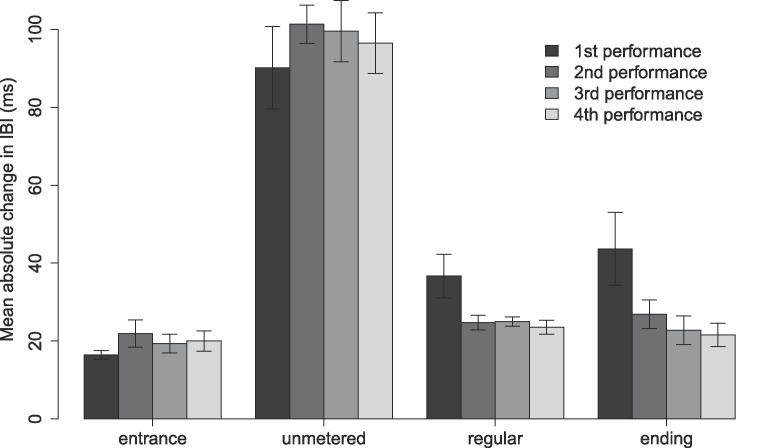
Absolute average change in IBI per piece section and performance. Error bars indicate standard error.

Linear mixed effects modelling (LME) was used to evaluate the effects of piece section and rehearsal time (including performances 1–4) on mean absolute differenced IBIs. Rehearsal time nested within duos was included as a random effect to account for repeated measures (Fig. 5). The effect of piece section was significant, $F(3,125)=225.56,p<.001,\eta^{2}=0.82$, but the effect of rehearsal time and the interaction were not (both p>.05).

Post-hoc tests (using a Bonferroni-adjusted α=0.008) indicated that mean absolute differenced IBIs were higher in the unmetered section than in the entrance, $t(124)=22.86,p<.001,\eta^{2}=0.81$, regular, $t(124)=20.48,p<.001,\eta^{2}=0.77$, and ending sections, $t(124)=19.72,p<.001,\eta^{2}=76$. Means in the regular section did not differ from the ending or entrance, and the ending and entrance did not differ from each other. Thus, tempo variability was highest in the unmetered section of the piece.

LME was likewise used to test the effects of piece structure and rehearsal time on median absolute primo-secondo asynchronies, with rehearsal time nested within duos included as a random effect. The effects of piece structure, $F(3,406)=34.05,p<.001,\eta^{2}=0.19$, and rehearsal time, $F(3,29)=5.00,p=.006,\eta^{2}=0.03$, were significant, but the interaction was not (p>.05).

Post-hoc tests (at a Bonferroni-adjusted α=0.008) showed higher asynchronies in the unmetered section than in the entrance, $t(406)=5.8,p<.001,\eta^{2}=0.08$, regular, $t(406)=10.0,p<.001,\eta^{2}=0.20$, and ending sections, $t(406)=6.2,p<.001,\eta^{2}=0.09$. Asynchronies in the ending did not differ from either the entrance or regular sections (all p>.008), and the entrance did not differ from the regular sections.

The main effect of rehearsal time was unsurprising, as synchronization can be assumed to improve quickly during the early stages of rehearsal. Of particular interest was the potential difference in median absolute asynchronies achieved in the third and fourth performances, which were completed with and without visual contact, respectively. No effect of visual contact was observed, t(27)=0.04,p=1.0 (asynchrony with visual contact M=44.1 ms, SD=40.8; without visual contact M=45.0 ms, SD=39.5).

The increased tempo variability and note asynchrony that occurred in the unmetered section of the piece confirms our expectation that timing would be less stable during this passage. In the following sections, we test for effects of piece structure on eye gaze behaviour with the expectation that this section in particular will prompt visual interaction between performers.

### 3.2 Effects of piece structure, rehearsal time, and instrument on unidirectional partner-directed eye gaze

**Fig. 6:**
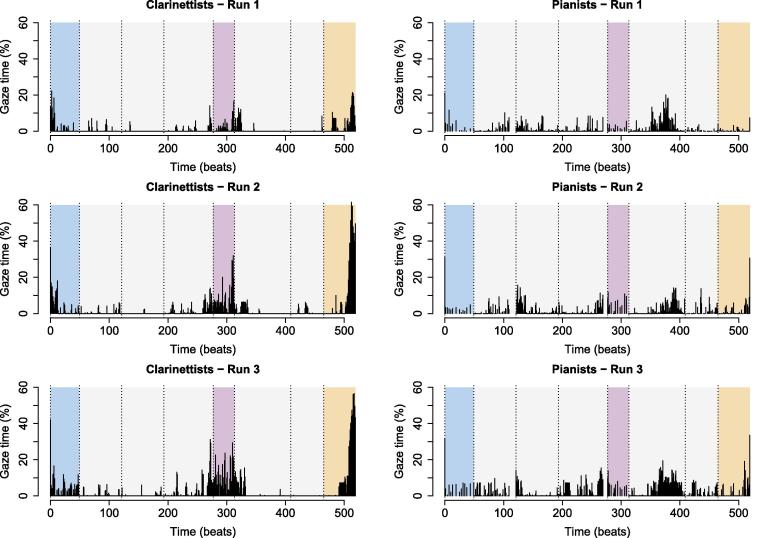
Unidirectional gaze per beat. Solid vertical bars indicate the percentage of each beat during which unidirectional gaze occurred. Beat “0” refers to the 3 s prior to piece onset, and beat 519 (the final beat) includes the 3 s after the final note onset. All other graph features are as described for Fig. 4.

Fig. 6 shows the percentage of time per beat, averaged across individual performers, that gaze vectors intersected with a co-performer’s face, body, or instrument – that is, the average percentage of time for which unidirectional partner-directed gaze occurred. Results are given separately for clarinettists and pianists, as some differences were observed between instrument groups (see below).

**Fig. 7:**
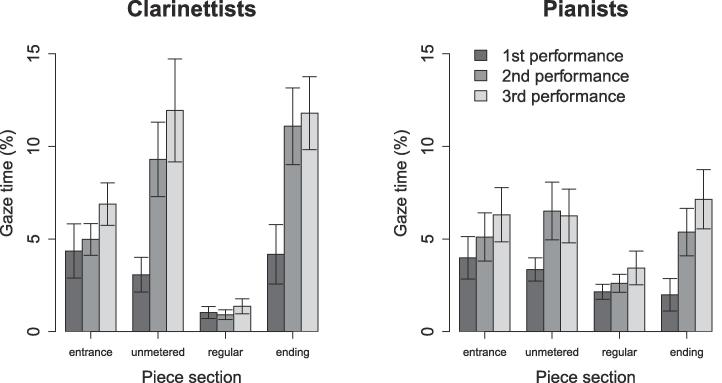
Unidirectional eye gaze across piece sections and performances: effects of piece section and rehearsal time on the percentage of time that musicians spent watching their partners. Error bars indicate standard error.

LME was conducted to test the effects of piece section, rehearsal time, and instrument on partner-directed gaze time, with rehearsal time nested within subjects included as a random effect to account for repeated measures. This yielded significant effects of piece section, $F(3,335)=23.83,p<.001,\eta^{2}=0.14$, and rehearsal time, $F(2,95)=15.91,p<.001,\eta^{2}=0.06$, and significant interactions between piece section and rehearsal time, $F(6,335)=3.36,p=.003,\eta^{2}=0.04$, and piece section and instrument, $F(3,335)=7.38,p<.001,\eta^{2}=0.05$ (Fig. 7). The main effect of instrument and other interactions were non-significant (all p>.05).

Post-hoc tests indicated that performers watched their partners a significantly larger percentage of the time in the entrance, $t(335)=5.0,p<.001,\eta^{2}=0.07$, unmetered, $t(335)=7.2,p<.001l,\eta^{2}=0.13$, and ending sections, $t(335)=7.5,p<.001,\eta^{2}=0.14$, than during the regular sections (significance evaluated at a Bonferroni-adjusted α=0.008). They also looked more at their partners during the 2nd, $t(91)=4.4,p<.001,\eta^{2}=0.18$, and 3rd performances, $t(56)=5.5,p<.001,\eta^{2}=0.35$, than during the 1st performance (evaluated at α=0.02). Within instrument groups, no between-section difference reached significance for pianists (evaluated at α=0.008; all p>.008), but clarinettists watched their partners more in the entrance, $t(335)=4.5,p<.001,\eta^{2}=0.06$, free, $t(335)=7.4,p<.001,\eta^{2}=0.14$, and ending sections, $t(335)=8.3,p<.001,\eta^{2}=0.17$, than in the regular sections, and more in the ending section than in the entrance, $t(335)=3.8,p=.004,\eta^{2}=0.04$ (evaluated at α=0.008). Within-section differences between pianists and clarinettists did not meet significance (evaluated at α=0.01; all p>.01).

Thus, performers spent more time looking at each other during periods of temporal instability than during periods of regular timing, in line with the hypothesis that uncertainty about co-performers’ intended timing would prompt visual interaction. On the other hand, they spent more time watching each other after rehearsing than before, indicating an increase in visual interaction once they had established shared intentions regarding how the piece should sound. Differences between sections were stronger for clarinettists than for pianists.

### 3.3 Effects of leader/follower roles on unidirectional partner-directed eye gaze

Sections of the piece in which leader/follower roles were implied by a melody/accompaniment structure were identified a priori. Primos led a total of 27 bars, distributed across entrance (4 bars), unmetered (half of the section, counted as 1 bar), regular (12), and ending (10 bars) sections, while secondos led a total of 14 bars, distributed across entrance (4 bars), unmetered (half of the section, again counted as 1 bar), regular (1 bar), and ending (8 bars) sections.

**Fig. 8:**
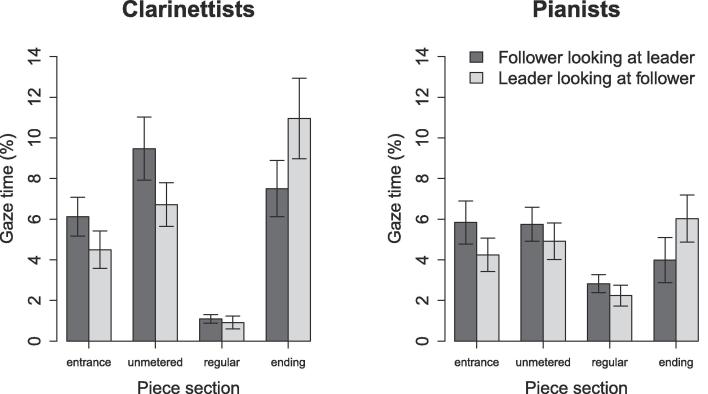
Unidirectional eye gaze across piece sections: effects of leader/follower roles and piece section on the proportion of time that musicians spent watching their partners. Error bars indicate standard error.

LME was used to test the effects of leader/follower role, piece section, and instrument on partner-directed gaze time (Fig. 8). The effect of piece section, $F(3,638)=31.8,p<.001,\eta^{2}=0.12$, and the interaction between piece section and instrument, $F(3,638)=7.66,p<.001,\eta^{2}=0.03$, were significant. The effects of leader/follower role and instrument and the other interactions were all nonsignificant (p>.05). Thus, the leader/follower roles that were implied by the score did not reliably influence patterns of partner-directed gaze.

### 3.4 Effects of piece structure and rehearsal time on face- versus body/instrument-directed eye gaze among clarinettists

**Fig. 9:**
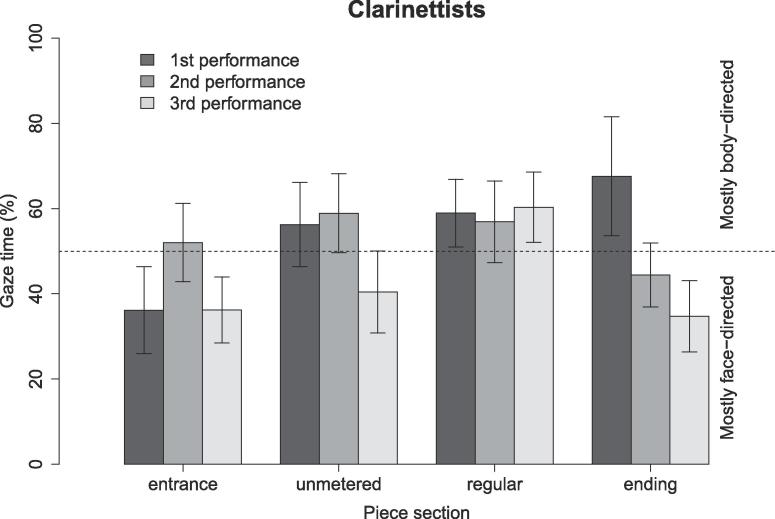
Unidirectional eye gaze towards clarinettists’ faces vs. bodies as a percentage of total partner-directed gaze time: effects of piece section and rehearsal time on the percentage of partner-directed gaze time for which body-directed gaze occurred. The horizontal dotted line indicates the proportion for which time spent watching the face and body/instrument would be equal. Error bars indicate standard error.

Pianists in this experiment were only able to see each other’s faces over the top of their music stands, but clarinettists were standing and able (at least partially) to see each other’s upper bodies and instruments. So, for clarinettists, we tested for differences in how much time they spent looking at their partner’s face versus their partner’s body/instrument (Fig. 9).

With data pooled across piece sections and performances, face- and body/instrument-directed gaze times were similar, t(163)=0.2,p=.84. LME was used to test for effects of section and rehearsal time on the percentage of partner-directed gaze samples for which the gaze vector intersected the partner’s face rather than body/instrument. This yielded a significant effect of piece section, $F(3,106)=4.33,p=.006,\eta^{2}=0.09$, and a significant interaction between piece section and rehearsal time, $F(6,105)=3.49,p=.003,\eta^{2}=0.14$. The effect of rehearsal time was non-significant (p>.05).

Post-hoc tests indicated that gaze in the entrance section was significantly more face-directed than gaze in the regular sections, $t(105)=3.5,p=.004,\eta^{2}=0.11$ (evaluated at a Bonferroni-adjusted α=0.008). No other between-section differences reached significance. Likewise, none of the pairwise comparisons between levels of piece section and rehearsal time reached significance (evaluated at α=0.02 for within piece sections/between performances; α=0.008 for within performances/between piece sections). Thus, clarinettists tended to focus more on their partner’s faces in the entrance than in regular sections.

### 3.5 Effects of piece structure, rehearsal time, and instrument on bidirectional partner-directed eye gaze

**Fig. 10:**
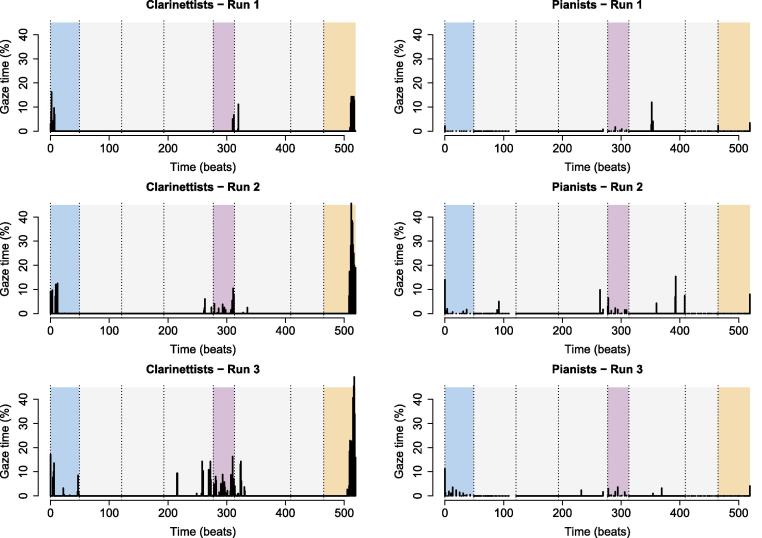
Bidirectional gaze per beat. Solid vertical lines indicate the proportion of each beat during which bidirectional gaze occurred. Beat “0” refers to the 3 s prior to piece onset, and beat 519 (the final beat) includes the 3 s after the final note onset. All other graph features are as described for Fig. 4.

Fig. 10 shows the average percentage of time per beat that both performers’ gaze vectors simultaneously intersected each other’s face, body, or instrument – the average percentage of time for which bidirectional partner-directed gaze occurred.

**Fig. 11:**
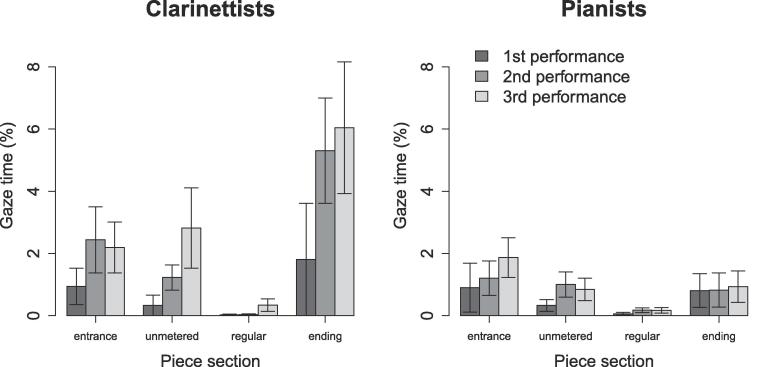
Bidirectional eye gaze across piece sections: effects of piece section and rehearsal time on the percentage of time that musicians spent watching each other. Error bars indicate standard error.

Bidirectional gaze occurred much less frequently than did unidirectional gaze, t(603)=9.6,p<.001,d=0.62. LME was used to test whether the same effects of piece section, rehearsal time, and instrument would emerge as for unidirectional gaze (Fig. 11). Rehearsal time nested within duos was included in the model as a random effect. The effects of piece structure, $F(3,371)=23.57,p<.001,\eta^{2}=0.14$, rehearsal time, $F(2,56)=8.69,p<.001,\eta^{2}=0.03$, and instrument, $F(1,14)=8.77,p=.01,\eta^{2}=0.02$, were significant, and there was a significant interaction between piece section and instrument, $F(3,371)=9.05,p<.001,\eta^{2}=0.05$. The effect of instrument and other interactions were non-significant (all p>.05).

Post-hoc tests indicated that performers spent more time watching each other during the entrance, $t(371)=5.3,p<.001,\eta^{2}=0.07$, unmetered, $t(371)=3.9,p<.001,\eta^{2}=0.04$, and ending sections, $t(371)=7.3,p<.001,\eta^{2}=0.12$, than during the regular section, and more time watching each other during the ending than during the unmetered section, $t(371)=3.8,p=.001,\eta^{2}=0.04$ (significance evaluated at a Bonferroni-adjusted α=0.008). Likewise, they spent more time watching each other during the 2nd performance, $t(120)=3.5,p=.002,\eta^{2}=0.09$, and 3rd performance, $t(31)=3.8,p=.002,\eta^{2}=0.32$, than during the 1st performance. Clarinettists watched each other more than pianists in the ending section, $t(116)=4.97,p<.001,\eta^{2}=0.17$ (evaluated at α=0.01). Clarinettists also watched each other more in the entrance, $t(371)=5.0,p<.001\eta^{2}=0.06$, unmetered, $t(371)=3.8,p=.004,\eta^{2}=0.04$, and ending sections, $t(371)=9.4,p<.001,\eta^{2}=0.19$, than in regular sections, and more in the ending than in the entrance, $t(371)=5.0,p<.001,\eta^{2}=0.06$, and unmetered sections, $t(371)=5.8,p<.001,\eta^{2}=0.08$.

Thus, bidirectional gaze occurred rarely, but varied according to piece structure and rehearsal time similarly to unidirectional gaze – occurring more often during moments of temporal instability, and increasing as performers became more certain of how they wanted the piece to sound. As was the case for unidirectional gaze, differences between piece sections were greater for clarinettists than for pianists.

### 3.6 Gaze behaviour and note synchrony

We tested whether increased visual interaction might improve note synchrony at piece onset, but no relationship was found between the proportion of the 3 s prior to piece onset that performers spent looking at their partners (i.e., unidirectional gaze time), and synchronization success, r=0.04.

### 3.7 Gaze behaviour and individual differences

All of our participants were highly trained professional musicians, but we considered the possibility that gaze patterns change as a function of musical expertise. Self-reported years of formal training and number of performances in the past year were tested, but neither shared a meaningful relationship with unidirectional gaze behaviour, operationalized as the proportion of performance time during which unidirectional gaze occurred, averaged across runs per participant (years of training, r=-0.08; number of performances, r=0.02).

## 4 Discussion

This study assessed the patterns of unidirectional and bidirectional eye gaze that occurred during duo performance of an unfamiliar piece, before and after brief periods of rehearsal. Piano and clarinet duos recorded four performances of a piece – one before, one partway through, and two at the end of a 20–40 min rehearsal session. Piece structure and rehearsal time were found to influence the percentage of performance time during which uni- and bidirectional gaze occurred. Specifically, musicians looked more often at their partners during periods of temporal instability than during periods of regular timing, suggesting the use of intention-driven gaze. They also spent more time watching each other after rehearsing than before, suggesting an increasing role for engagement-driven gaze as they learned the music. Clarinettists, finally, exhibited a preference for face-directed rather than body-directed gaze during the entrance section of the piece. Each of these observations is discussed in greater detail below.

One of our central hypotheses regarding intention-driven gaze was that moments of temporal instability would prompt performers to interact visually, and this hypothesis was confirmed. Performers spent a greater percentage of their time watching each other during the unmetered section than during regularly-timed sections. This finding is in line with the idea that ensemble musicians exchange visual signals when temporal coordination is threatened by ambiguity in the timing information given by a musical score (Bishop and Goebl, 2015, Kawase, 2014a, Kawase, 2014b). More broadly, it aligns with research suggesting that, on interpersonal coordination tasks, people draw flexibly on information from different modalities, depending on which modality seems most reliable at a given moment (Elliott et al., 2010, Hove et al., 2013).

During the unmetered section of the piece, temporal ambiguity came from both the absence of a notated meter – which encouraged high note-to-note variability in tempo – and the presence of long held notes, including the notes with fermatas shown in Fig. 1. As a side note, we should mention that several (though not all) duos deliberately integrated visual cues into their performance routine during this section of the piece, following verbal discussion (e.g., “watch me here, and I’ll give the cue”). This observation is in line with findings reported by Williamon and Davidson (2002), who also found musicians to deliberately integrate visual cues into a performance plan in this way.

During the entrance section, the primary instance of temporal ambiguity came at the moment of piece onset, which the primo and secondo were intended to synchronize. Both uni- and bidirectional gaze peaked during the seconds just prior to piece onset. On one hand, it is unsurprising that performers watched each other closely as they prepared to coordinate the first chord of the piece, given the absence of prior audio. On the other hand, it is interesting to note the peak in bidirectional gaze at this point. A peak in uni- but not bidirectional gaze would have suggested that one performer visually monitored the other for information about when to begin. The peak in bidirectional gaze that we observed instead suggests that performers monitor each other. In doing so they may exchange a range of information about their shared focus of attention, their joint willingness to interact, and their intended timing of the first notes of the piece.

Uni- and bidirectional gaze both also peaked in the beats leading up to the final chord of the piece. This was particularly the case for clarinettists. Many duos gradually slowed their tempo in the last notes of the piece, employing the well-known expressive device referred to as the “final ritard” (Friberg and Sundberg, 1999, Honing, 2003), but overall, there was less temporal ambiguity in this section of the score than, for example, in the unmetered section. We would therefore suggest that the increase in visual interaction at the end of the piece reflects performers’ attempts to monitor each other’s attention, rather than exchange specific information about their intended timing.

For pianists, there was also an unexpected uptake in unidirectional gaze during one of the regularly timed sections of the piece. This uptake occurred in the third bar (53) of the line shown in Fig. 1b (around beat 380 in Fig. 6, Fig. 10). It seems unlikely that performers would be prompted to look to each other for timing cues during this passage, as the timing is relatively unambiguous. Rather, we would speculate that the uptake in partner-directed gaze relates to the positioning of the passage on the score: this line appeared at the top of the page that stood in the center of the music stand, only a short vertical distance away from the co-performer’s face. It would have been easier for performers to glance at their partners while playing this line than while playing most other parts of the piece, which would have required the eyes to traverse a greater distance.

The piano score spanned six pages, which we fixed together as two groups of three, so that there was only one page turn at the end of page 3. Beat 380 fell at the top center of page 5. A corresponding, though smaller, uptake can be seen at the start of the second regularly-timed section on score page 2 (see third dotted vertical line in Fig. 6), which likewise fell at the top center of the music stand, though this uptake could also be attributed to the change in meter that occurs at this spot. Uptakes in partner-directed gaze did not occur for clarinettists at either location, likely because they were free to reposition themselves (since they were standing), and often ended up at an angle rather than facing each other directly (as in Fig. 3b). The fact that pianists chose to glance at their partner when it became easier to do so is telling – it suggests a propensity to engage visually even when coordination does not depend on it.

We also tested the hypothesis that performers would look less at their partners after rehearsing than before. This is what we would expect to see if visual interaction were primarily a means of communicating individual intentions. Performers’ familiarity with each other’s intentions would increase as they established a shared interpretation of the piece, reducing their reliance on visual cues. Challenging this hypothesis, however, the results showed an increase in partner-directed gaze with rehearsal. It seems that performers chose to engage visually with each other once they were familiar enough with the score to be able to glance away from it. Note synchronization was maintained in the final performance, even though performers could not see each other, indicating that duos do not require visual contact in order to coordinate temporally – in line with prior research (e.g., Bishop and Goebl, 2015, Fulford et al., 2018). The increase in partner-directed gaze that occurred across the first three performances thus indicates a desire to interact visually when doing so is not necessary to maintain coordination. Such a finding is in line with the hypothesis that gaze can be engagement-driven – that musicians interact visually in order to monitor each other’s attention and engagement. It might also be that performers seek additional means of interaction to ensure coordination once they have specific expectations for how the piece should sound, and what each performer should contribute.

For clarinettists, we found a preference for face- over body-directed gaze only in the entrance section of the piece. We had hypothesized that clarinettists would spend more time looking at each other’s faces than each other’s bodies if their aim was to monitor each other’s facial expressions and gaze direction; in contrast, greater focus on the body/instrument might indicate an attempt to monitor gestures involved in sound-production, including fingering and breathing. Our observations suggest that clarinettists were compelled to monitor each other’s expressions and attention as they coordinated the entrance of the piece. The lack of meaningful results in other conditions could be partially attributable to imprecision in the identification of gaze vectors that intersected near the borders of the face and instrument regions. A possible consequence is that performers who were watching their partner’s instrument close to the mouthpiece might sometimes have been categorized as watching their partner’s face. The best fix to this issue would have been periodic recalibration of the glasses throughout the 1-h recording session.

Another likely explanation is the way clarinettists were positioned in the recording space in relation to each other. Their view of each other was partially blocked by the music stand and score, and depending on how they chose to stand (e.g., close to the music stand vs. further away; facing their partner directly vs. at an angle), how high they had adjusted the music stand, how tall they were compared to their partner, and how much they and their partner moved, their views of each other were sometimes (variably, throughout the performance) partially occluded. We chose to prioritize naturalistic playing conditions by giving performers some freedom of movement, but a more controlled recording setting might yield a clearer identification of which cues (face or body) clarinettists attend to while performing.

We had hypothesized that performers might spend more time watching their partner when playing accompaniment passages (following) than when playing melody passages (leading). This hypothesis was not confirmed; partner-directed gaze was not reliably influenced by the leader/follower roles implied by melody/accompaniment relationships in the score (though there was a nonsignificant tendency for performers to watch their partners more when following than when leading, in all but the ending section). This lack of effect is in contrast to the idea that followers visually monitor leaders for cues indicating how to play. Instead, it seems that both leaders and followers have reason to watch each other.

The lack of effect here is at odds with prior research that has shown followers to look more towards leaders than vice versa (Kawase, 2014a, Kawase, 2014b), possibly because of differences in experimental design. Kawase’s participants were assigned leader and follower roles, while our participants were not given any explicit instruction regarding who should lead or follow. We were instead interested in whether gaze patterns would be affected by the leader/follower relationships that emerge naturally during duo performance, with the acknowledgement that these relationships vary within and between performances and are influenced by numerous factors, including piece structure, performance conventions, and the social and personality dynamics that arise between the performers. Future research might investigate whether some of these other factors have a stronger influence on gaze behaviour.

It is important to acknowledge limits to the generalizability of our results. We tested a specific type of performance situation: participants were rehearsing, not performing for an audience; the music was unfamiliar, not previously practiced; and participants were positioned in the recording space in a way that encouraged visual contact. Changes to any one of these factors would likely influence gaze behaviour. Skilled musicians are flexible in their abilities to draw cues from their co-performers’ audio/visual signals, and readily adapt to changes in playing conditions (Bishop and Goebl, 2015, Glowinski et al., 2016). As such, there is no single pattern of interactive or communicative behaviours that could be said to enable duo performance across different playing conditions. The patterns of gaze behaviour that were observed here are interesting primarily because of what they imply about how musical interaction unfolds. Our results imply that duo musicians who have full access to each other’s audio signals are prompted to supplement their audio interaction with interaction via a second modality (vision) when they want confirmation of piece timing or their partner’s focus. Future research might investigate how interaction techniques differ across performance conditions.

The design of this experiment rests on the assumption that musicians who are learning a new piece from the score will have to focus their visual attention on the score most of the time. The fact that they have few opportunities to look elsewhere makes their partner-directed glances meaningful – they would not divert their gaze away from the music unless they had reason to do so. Without such a demand on their visual attention (e.g., if the music were memorized or the task was to improvise), it would not be possible for us as experimenters to deduce meaning from their partner-directed gaze. In this paper, we have attributed meaning to participants’ partner-directed glances according to the conditions in which they occur. First, we have argued that partner-directed glances occurring in moments of potential temporal variability likely reflect an effort to confirm shared expectations regarding performance timing. In such moments, performers may be watching for cues indicating their partner’s intended timing. In interpreting some of our other findings, we have suggested that performers keep track of each other’s attention and engagement by visually monitoring gaze direction, body movements, and facial expressions. The frequency of bidirectional gaze and the increase in partner-directed gaze that occurred with rehearsal were observations that we took as evidence that performers choose to visually monitor each other even when coordination is not threatened by uncertainty over timing.

### 4.1 Conclusions

The results of this study suggest that duo musicians seek visual interaction with their co-performer, particularly – but not exclusively – when coordination is threatened by temporal instability. Performers seem to monitor each other for timing cues when uncertain of whether temporal coordination will be successful. Visual interaction also increases as performers grow more familiar with the piece, which may reflect an attempt to engage with each other in a way that enables higher levels of collaboration.

Gestural cues are an important source of information as performers interact visually, but a characterization of performers’ communicative gestures is still missing from the literature. Our ongoing investigation of the motion data collected as part of this study examines how performers move when they know that a co-performer is watching, where in the performance explicit cueing gestures are given, and how performance gestures evolve across rehearsals.

In the literature, it has been hypothesized that emergent coordination between ensemble members is supported by low-level sensory couplings (e.g., Maes, 2016). Visually monitoring co-performers should facilitate such couplings by relaying information about body movement and facial expressions. Furthermore, performers who are able to visually confirm each other’s attention and engagement might feel more engaged in the collaborative task themselves, and perhaps more willing to take creative risks. Along these lines, an interesting continuation of this research would be to test the contribution of visual interaction to the quality of ensemble musicians’ performance experiences and, importantly, to the occurrence of group flow. The term group flow describes an intrinsically-rewarding state of intense focus, effortlessness, and perceived success that is shared among collaborating members of a group (Cochrane, 2017, Hart and Di Blasi, 2015, Sawyer, 2006). Reports from ensemble musicians suggest that a feeling of deep connectedness between co-performers is critical to group flow (Hart & Di Blasi, 2015). Visual interaction could help to support such feelings of connectedness.

## Figures and Tables

**Figure:**
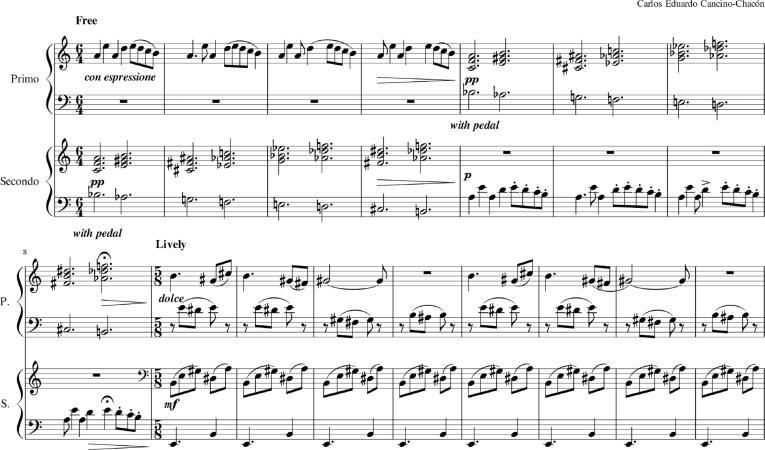


**Figure:**
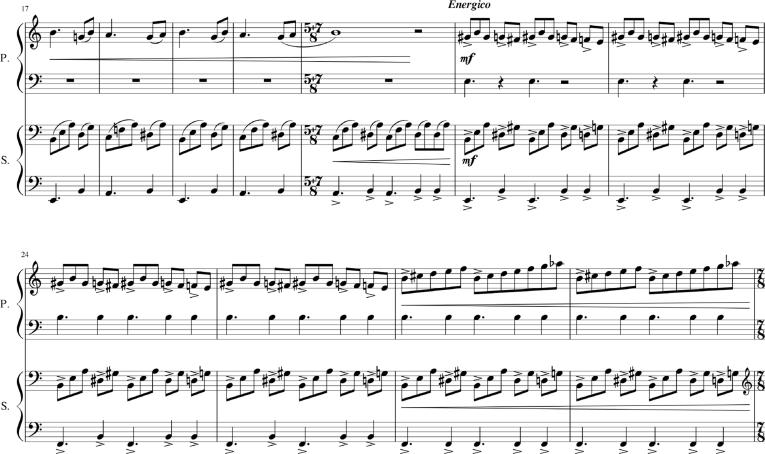


**Figure:**
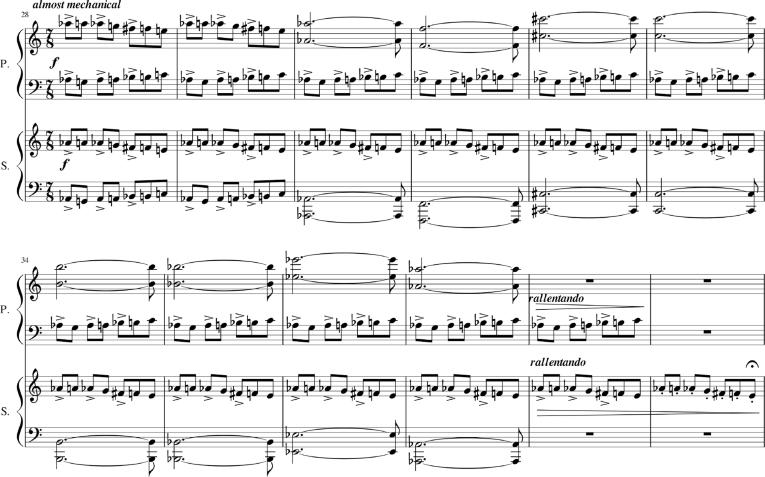


**Figure:**
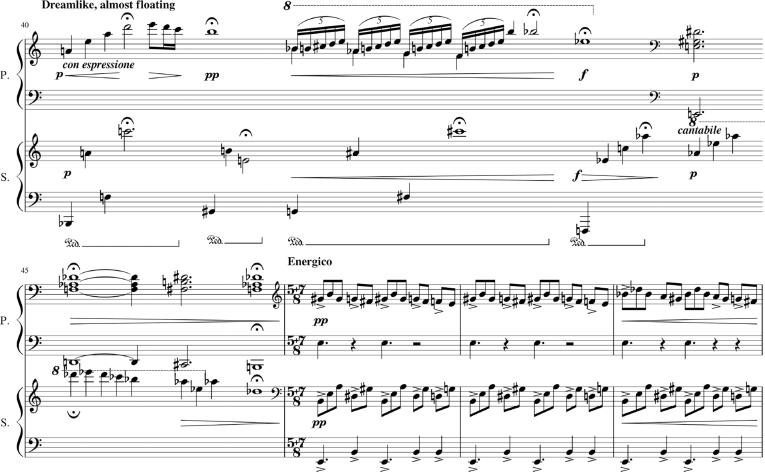


**Figure:**
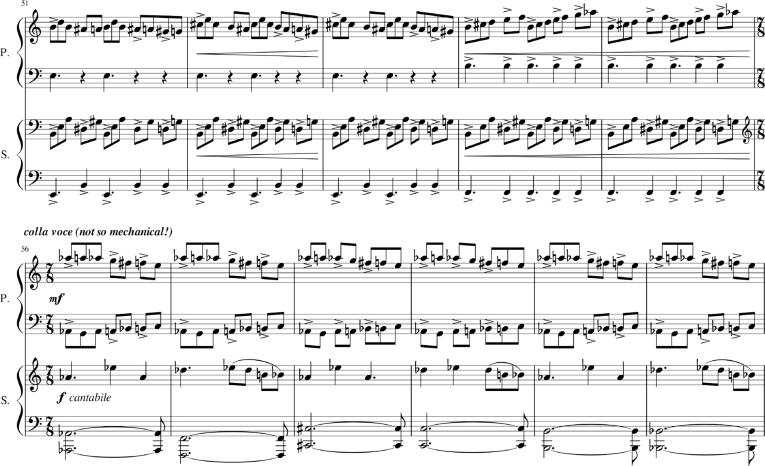


**Figure:**